# Keynote 2

**DOI:** 10.1093/eurpub/ckac092.002

**Published:** 2022-08-29

## Abstract

**Title of HEPA 2022 Keynote presentation:**

Physical activity, air pollution, and much more: the case for systems thinking in urban policies.

**Description of HEPA 2022 Keynote presentation:**

Physical activity may be one of greatest benefits of active travel -however there are many more, and in some cases there could be some trade-offs too. Yet, we create disjointed targets, campaigns and regulations to tackle in siloed and often piecemeal approaches major urban health and environmental challenges such as climate change, physical inactivity, air pollution, traffic injuries, social isolation, stress, inequalities, etc. The way we address these problems matters, and this lack of joined up thinking is a wasted opportunity. If we let the promotion of a healthier, more sustainable and resilient society be the goal of our policies rather than merely compliance with singularly focused standards and targets, we will find different types of solutions emerge as most advantageous. For example, in addition to providing a seamless way to integrate physical activity into daily routines, walking and cycling can be a convenient and low-cost alternatives to driving, thus reducing air pollution, green house gas emissions and noise; the infrastructure and urban design features conducive to walking and cycling may in turn have further benefits, such as traffic safety and greenspace exposure; etc. Evidence on benefits, co-benefits, and trade-offs of different types of urban policies that tackle some of our greatest urban health challenges jointly will be presented, making the case for holistic approaches to urban policy decision making, particularly through urban planning and design strategies. We will end by discussing avenues for greater integration of health in urban decision making.

